# Addressing uncertainties in correlative imaging of exogenous particles with the tissue microanatomy with synchronous imaging strategies

**DOI:** 10.1093/mtomcs/mfad030

**Published:** 2023-05-16

**Authors:** Alexander P Morrell, Richard A Martin, Helen M Roberts, Hiram Castillo-Michel, J Frederick W Mosselmans, Kalotina Geraki, Adrian T Warfield, Paul Lingor, Wasif Qayyum, Daniel Graf, Maria Febbraio, Owen Addison

**Affiliations:** Faculty of Dentistry, Oral & Craniofacial Science, Kings College London, Guy's Hospital, London, UK; Aston Institute of Materials Research, Aston University, Aston Triangle, Birmingham, UK; Aston Institute of Materials Research, Aston University, Aston Triangle, Birmingham, UK; Oral Health Clinical & Translational Research Centre, University of Alberta, Edmonton, Canada; European Synchrotron Radiation Facility, Grenoble, France; Diamond Light Source, Didcot, UK; Diamond Light Source, Didcot, UK; University Hospitals Birmingham NHS Foundation Trust, Birmingham, UK; Department of Neurology, School of Medicine, Technical University of Munich, Klinikum rechts der Isar, Munich, Germany; Oral Health Clinical & Translational Research Centre, University of Alberta, Edmonton, Canada; Oral Health Clinical & Translational Research Centre, University of Alberta, Edmonton, Canada; Oral Health Clinical & Translational Research Centre, University of Alberta, Edmonton, Canada; Faculty of Dentistry, Oral & Craniofacial Science, Kings College London, Guy's Hospital, London, UK; Oral Health Clinical & Translational Research Centre, University of Alberta, Edmonton, Canada

**Keywords:** synchrotron X-ray fluorescence spectroscopy, correlative imaging, exogenous metal imaging, endogenous elemental imaging, metal-labelled antibodies, lanthanide X-ray fluorescence, synchrotron confocal X-ray fluorescence spectroscopy

## Abstract

Exposure to exogenous particles is of increasing concern to human health. Characterizing the concentrations, chemical species, distribution, and involvement of the stimulus with the tissue microanatomy is essential in understanding the associated biological response. However, no single imaging technique can interrogate all these features at once, which confounds and limits correlative analyses. Developments of synchronous imaging strategies, allowing multiple features to be identified simultaneously, are essential to assess spatial relationships between these key features with greater confidence. Here, we present data to first highlight complications of correlative analysis between the tissue microanatomy and elemental composition associated with imaging serial tissue sections. This is achieved by assessing both the cellular and elemental distributions in three-dimensional space using optical microscopy on serial sections and confocal X-ray fluorescence spectroscopy on bulk samples, respectively. We propose a new imaging strategy using lanthanide-tagged antibodies with X-ray fluorescence spectroscopy. Using simulations, a series of lanthanide tags were identified as candidate labels for scenarios where tissue sections are imaged. The feasibility and value of the proposed approach are shown where an exposure of Ti was identified concurrently with CD45 positive cells at sub-cellular resolutions. Significant heterogeneity in the distribution of exogenous particles and cells can be present between immediately adjacent serial sections showing a clear need of synchronous imaging methods. The proposed approach enables elemental compositions to be correlated with the tissue microanatomy in a highly multiplexed and non-destructive manner at high spatial resolutions with the opportunity for subsequent guided analysis.

## Graphical Abstract

**Graphical Abstract:**
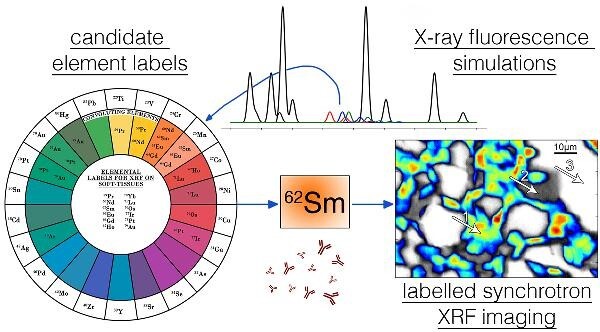
Candidate elements conjugated to antibodies were used to label biological tissues and have been imaged using X-ray fluorescence spectroscopy.

## Background

Exposure to exogenous particles through pulmonary exposures through inhalation,^1–3^ ingestion,^4^ surface adsorption,^5^ and theragnostic^6^ routes or due to deterioration of implanted biomaterials^7,8^ is of increasing concern to human health. While proving the presence of exogenous particles within physiological tissues and fluids is important, this only presents a partial picture in the assessment of the associated toxicity. Characterization of the chemical species, the spatial distribution, and the involvement of a stimulus with the tissue microanatomy are essential to understand the mechanisms underpinning the biological response.^9–11^ In a recent review, Bishop *et al*. discussed routine analytical approaches to identify the key characteristics of the tissue composition and the presence of common exogenous stimuli,^12^ including light^13^ and electron microscopy,^14^ X-ray [X-ray fluorescence spectroscopy (XRF),^15^ particle-induced X-ray emission (PIXE),^16^ etc.] and vibrational spectroscopy (Raman,^17^ FTIR,^18^ etc.), and mass spectrometry.^19^ However, no single technique can interrogate all features, and therefore multiple methods are routinely used requiring comparison of related but discrete samples, often in the form of adjacent tissue sections.^20^ Unfortunately, this measurement strategy has the potential to be confounded by any three-dimensional heterogeneity in tissue composition or in the distribution of the particle/fibre stimulus.

Routinely any three-dimensional complexity is simplified to two-dimensional (2D) tissue sections for histological and immunohistology analysis. Correlations are often described subjectively, but with the increased adoption of artificial intelligence and machine learning approaches, computer-assisted histopathology is becoming more routine.^21^ One analysis method that has significantly benefitted from these developments is the implementation of correlative algorithms to assess spatial relationships between features of interest. These analysis methods are often used to describe the position of cells and/or particle exposures within the tissue microanatomy, generating data about density and complexity of cellular compositions and their interactions with exogenous stimuli.^22^ However, such approaches must take into account uncertainties introduced by the use of serial tissue sections to avoid over- or misinterpretation of the data; nevertheless, this is infrequently considered.

Development of single synchronous imaging strategies that can identify all features of interest simultaneously on the same prepared sample would allow correlative analyses to be performed with improved confidence. This theoretically can be achieved by choosing an appropriate imaging approach to attain a high sensitivity for one or more features of interest(s) and supplementing the tissue sample with specific labels enabling previously undetectable features to be identified. This concept has been explored where antibodies have been conjugated to heavy metals, including gold and silver nanoparticles, thus allowing elemental imaging approaches to detect biological epitopes.^23^ More recently, the use of rare-earth metals as labels has significantly improved the multiplexed capabilities of elemental tags surpassing the restrictions of conventional fluorophores that are typically limited to four to six simultaneous tags. Lanthanide-conjugated antibodies coupled with isotopic-sensitive imaging probes (mass spectrometry) yield capabilities of imaging over 40 labels simultaneously in a technique known as imaging mass cytometry (IMC).^24^ IMC is a powerful technique for exploring fundamental mechanisms of cellular physiology especially in cancer and diabetes research where identification of several tags simultaneously offers a significant advantage.^25–27^ However, the ability to image and characterize the underlying elemental composition (endogenous and exogenous elements) concurrently with these labels is unexplored. Whilst triple-quad-based mass spectrometry approaches offer the highest sensitivity allowing dilute endogenous elements to be characterized, these are typically limited to detecting only a few isotopes simultaneously limiting the multiplexed nature.^28^ New time-of-flight technology specifically allowing full mass ranges to be imaged may play a pivotal part in simultaneous detection of endogenous elements and heavy-metal labels.^29^ Here, we present data to highlight complications of correlative analysis between the tissue microanatomy and elemental composition associated with imaging serial tissue sections. This is achieved by assessing both the cellular and elemental distributions in three-dimensional space using optical microscopy on serial sections and confocal X-ray fluorescence spectroscopy on bulk samples, respectively. We propose a new imaging strategy using lanthanide-tagged antibodies with XRF as opposed to mass spectrometry. XRF offers significant complementary advantages over a mass spectrometry probe, the fundamental difference being that samples are ablated in IMC, and therefore guided analysis cannot be conducted on the samples post-measurement. In contrast, XRF can be used to identify regions of interest allowing subsequent detailed interrogation.^30^ For example, X-ray absorption spectroscopy and X-ray scattering techniques can be used to define chemical speciation of elements and structural information within the specified region of interest.^31^ As understanding the chemical speciation of elements is essential for determining biological reactivity, this is a significant advantage of X-ray-based modalities over destructive mass spectrometry approaches.^7^ Mass filtering is routinely applied to IMC to improve detection sensitivity of lanthanides. This process prevents parallel detection of low-*Z* elements preventing subsequent correlative analyses between endogenous and exogenous elements. Here, we present the applicability of using lanthanide-tagged antibodies with XRF and provide a detailed report on the suitability of each tag and the difficulties associated with this promising approach.

## Results

### Three-dimensional heterogeneity in tissue microanatomy and in the distribution of exogenous particles

**Fig. 1:**
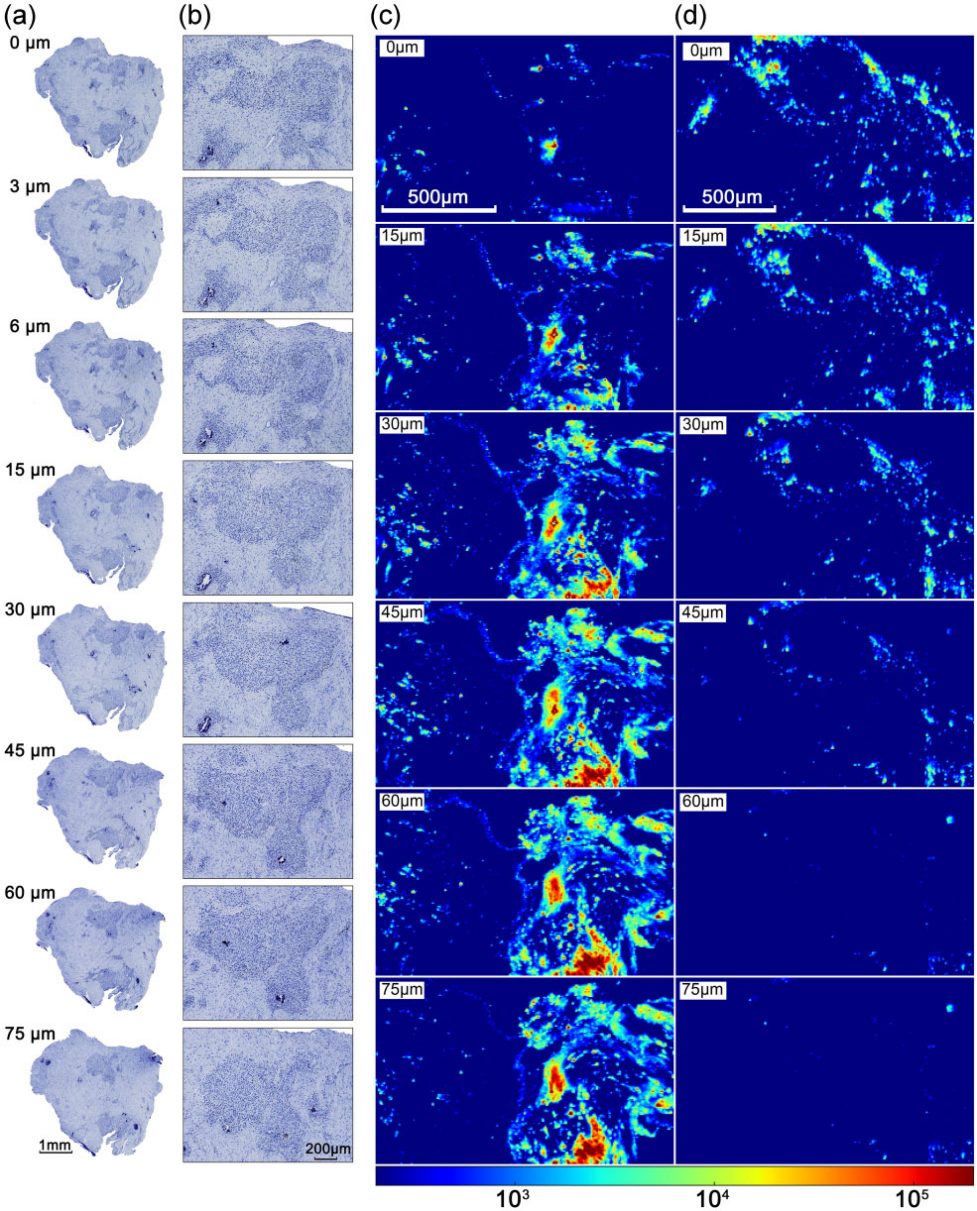
Demonstrates the three-dimensional variation in cellular content associated with serial histological sectioning (a and b) and Ti distribution (c and d) from tissues previously associated with a Ti BAH. (a) Tissues were prepared to 3 µm in thickness, counterstained with haematoxylin, and the depths of each section are listed accordingly. (b) Magnified inserts are displayed showing an inflammatory infiltrate. Confocal XRF images displaying the 3D Ti distribution within two tissue sections (c) and (d), which are equivalent tissues imaged in (a) and (b). The distributions were observed at depth increments of 15 µm and are labelled accordingly. The colour bar represents fluorescence Ti counts.

Figure 1 shows optical and confocal XRF images of tissue sections prepared from an inflamed soft human tissue previously associated with a failing Ti bone anchored hearing aid (BAH). In the optical image panel (Fig. 1a and b), the sections are immediately adjacent to each other at the top and bottom of the image are separated by 15 µm, which are labelled accordingly. A magnified insert is included showing the same region containing a mixed inflammatory cell infiltrate at each depth. The images show that larger cellular infiltrates (higher density of blue colour) persist throughout the entire volume interrogated, whereas smaller regions may be lost when comparing similar tissue sections taken at different depths. It is also clear that exact cell positions and cell density are variable even in immediately adjacent sections. The entire tissue section maintains largely the same morphology with the most variation around the circumference of the tissue, which is expected to be caused by the tissue processing. Alongside the changes in tissue microanatomy seen in the serial sections, the confocal XRF images (Fig. 1c and d) also show significant three-dimensional heterogeneity in the distribution of the exogenous elemental signal (Ti). At the level of the first tissue section (top of the image in Fig. 1c), only a small distribution of mostly isolated Ti particles is observed. This accumulation significantly increases moving inwards into the sample with large agglomerating Ti features >30 µm present within the sample. Contrastingly, the reverse pattern is observed in Fig. 1d. Similarly, to the cellular infiltrates, larger Ti features persist through multiple depths of tissue and smaller features appear unpredictably.

### Potential for correlating endogenous and exogenous XRF signals with tissue microanatomy using lanthanide labels

**Fig. 2:**
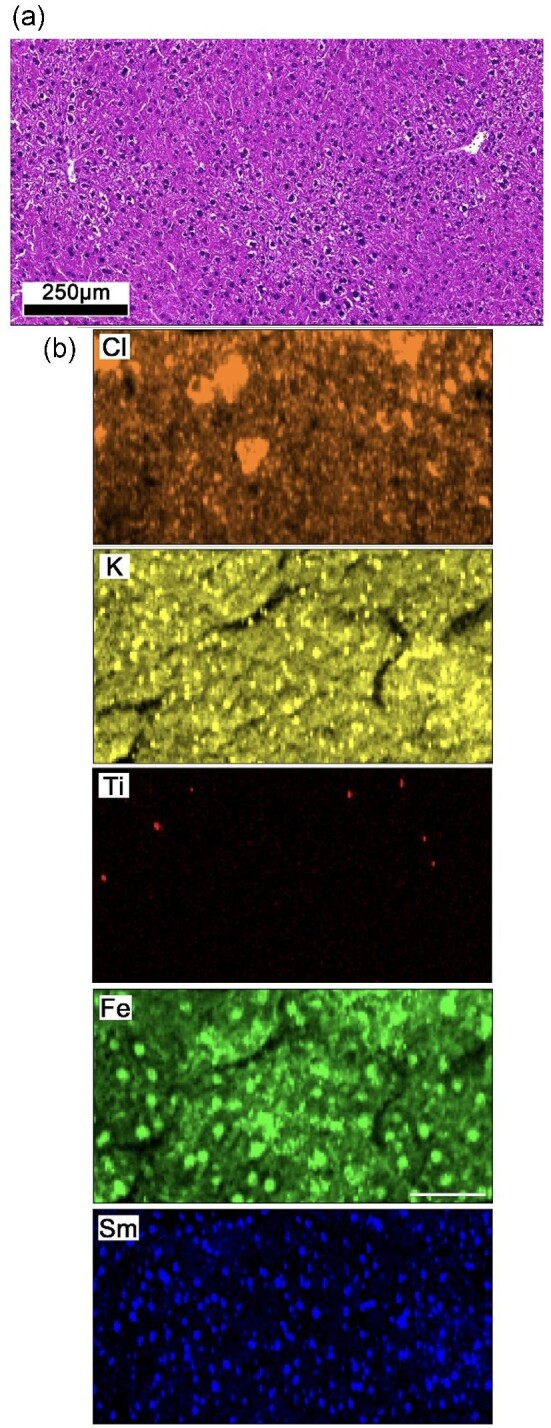
Analyses of serial murine liver sections. (a) H&E image identifies cellular compositions consisting of diffuse lymphocytes. (b) XRF image panel (1.5 µm resolution). H&E and XRF images do not represent identical regions as these were imaged on serial sections. Scale bar represents 50 µm.

Murine liver and spleen associated with animals subjected to a thioglycollate peritonitis model with a concomitant intraperitoneal Ti exposure were studied. This model generated a predictable and controlled inflammatory response caused by an injection of thioglycollate into peritoneum. The addition of Ti (anatase as nanoparticles) in saline served as the exogenous exposure. The exposure model was not the primary focus of this study, but tissues were available for secondary usage, allowing the investigators to avoid *de novo in vivo* animal experiments. In the acquired tissues, a diffuse distribution of lymphocytes is seen in H&E liver sections (Fig. 2a). An adjacent serial section was stained with a Sm CD45 conjugated antibody and imaged using XRF. A large area of tissue was initially measured using a defocussed beam (50 µm beam footprint) to identify areas of Sm concentration. Subsequently, a 1.5 µm beam was used to image the identified regions at sub-cellular resolution. XRF provided simultaneous detection of the endogenous elements (Cl, K, and Fe were chosen due to high signal-to-noise ratio), the exogenous exposure (Ti), and the Sm-label (Fig. 2b). The Cl and K signals align well with the tissue geometry, reveal non-specifically cell location, but provide poor contrast with the extra-cellular matrix (ECM). The strong Fe signal indicates the position of hepatocytes but cannot be used exclusively to define cell size and shape due to subjective segmentation and uncertainty introduced by inhomogeneous tissue thickness. Quantitative image analysis of cell size (*n* = 50 cells) and statistical testing using Pearson's correlation methods (*r* = 0.974, *P* < 0.001) confirmed a strong correlation between the dimensions of CD45 positive cells identified using the introduced Sm signal from XRF images and lymphocytes identified within serial H&E images.

**Fig. 3:**
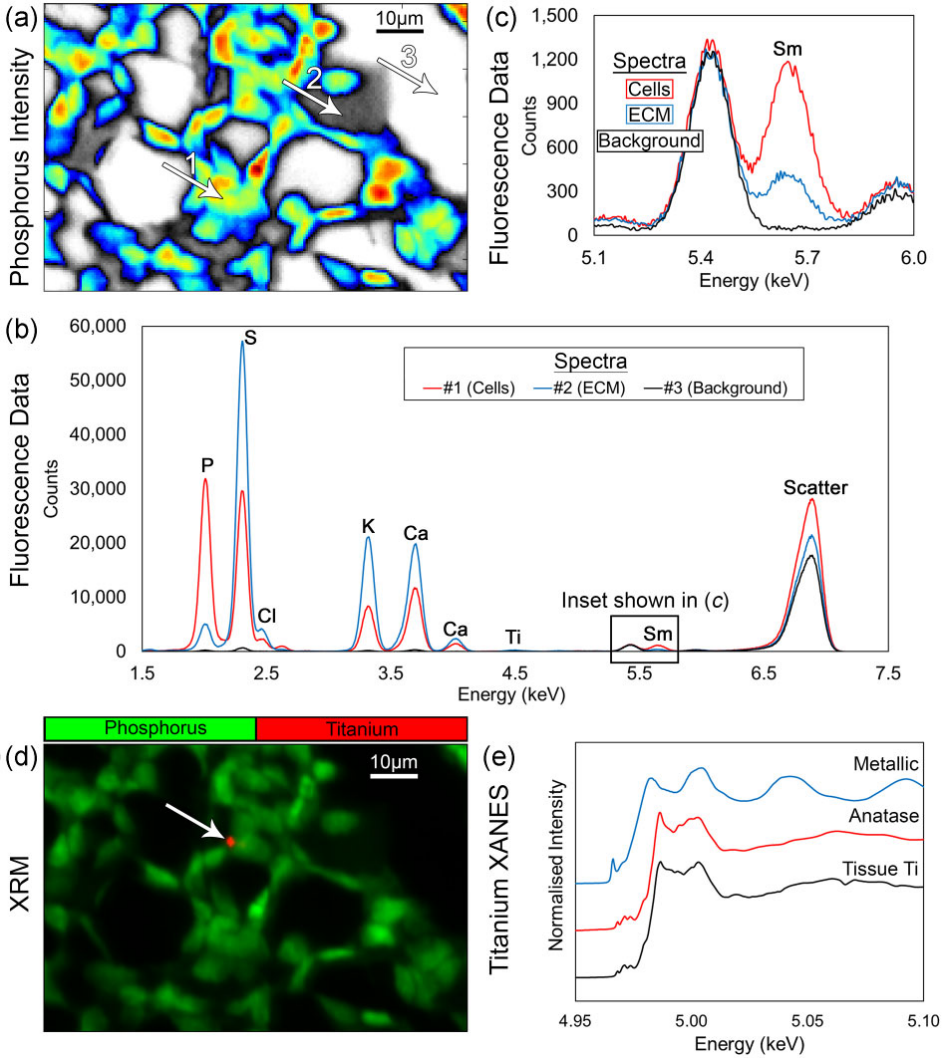
Analysis of a murine spleen, (a) P XRF intensity map segmented to show CD45 positive cell positions. (b) XRF spectra taken from a CD45 positive cell (red), ECM (blue) and background (black). (c) Zoomed in region within (b) to highlight the Sm peak. (d) XRF image showing the position of exogenous Ti (red) within the P image (green). (e) XANES analysis showing Ti feature highlighted in (d) with anatase and metallic Ti standards.

To demonstrate epitope-specific binding of the Sm-antibody, the position of CD45 positive cells was extracted from the P intensity image (murine spleen, Fig. 3a). Coloured regions represent cells with ECM and background in greyscale. A total of 98.1% of pixels identified as CD45 positive cells contained an Sm signal above background, demonstrating high sensitivity, whereas only 1.1% of regions recognized as background were Sm positive demonstrating high specificity. XRF spectra from CD45 positive cell (red), ECM (blue), and background (black) are shown in Fig. 3b, with an inset highlighting the distinct Sm peak in Fig. 3c. The peak present at ∼5.4 keV is caused by the emission of Cr, which likely originates from the excitation of peripheral detector casings. X-ray absorption near edge structure (XANES) was performed on an isolated Ti feature, highlighted in Fig. 3d, demonstrating the potential of chemical-guided analyses. The XANES spectrum, Fig. 3e, shows close resemblance to anatase (TiO_2_), which is consistent with the intraperitoneal exposure and demonstrates its systemic distribution.

## Discussion

Understanding the spatial relationship between cellular compositions and exogenous particles or fibres causing adverse tissue reactions is important to fully understand the mechanisms underpinning the biological response(s). Multiple imaging approaches are routinely used in combination to investigate this relationship, but often differing and incompatible sample preparations are required for each method to ensure an accurate measurement. To address this, investigators commonly correlate measurements taken on immediately adjacent tissue sections, allowing each to be processed according to the needs of the measurement technique deployed. However, the use of serial sections adds uncertainty in correlative analyses as tissue sectioning and processing risks modification of the feature(s) of interest. In addition, discrepancies in image resolution between each approach and the requirement of post-analytical alignment introduce error.

To study the natural variation of the tissue microanatomy and exogenous elements in three-dimensional, we investigated correlations between serial sections imaged using routine optical microscopy and bulk tissue containing exogenous metal particles with confocal XRF. The serial sections contained similar bulk morphologies throughout with the most variation observed around the circumference of the tissue, which is likely due to sectioning. For this reason, regions of interest were examined within the central parts of the tissue to reduce induced variation associated with tissue processing. No attenuation-based corrections on the confocal XRF data were performed, which would account for variable damping of Ti fluorescence at different tissue depths. These corrections require accurate compositional information of the path between the probed atom and the detector. Due to the heterogenous nature of Ti particles within the tissue, this information could not be attained. However, as three-dimensional distributions were observed where higher concentrations of Ti were deeper in the tissue, it is evident damping of the fluorescence X-rays is not preventing identification of these features at depths of 75 µm. Some Ti particles were visible within the optical images as black features, however, when compared with XRF data it is clear this is not a representative distribution. In Fig. 1, inflammatory lesions and accumulations of Ti present similar characteristics whereby larger features or clusters of features persist through a greater depth, whereas smaller isolated particles of Ti or single cells are highly variant through the depth of the tissue. The combined findings demonstrate that significant three-dimensional variation in both tissue microanatomy and the distribution of an exogenous particulate stimulus can occur at relatively short ranges, with the potential to introduce significant uncertainty in the interpretation of correlated 2D data.

**Fig. 4:**
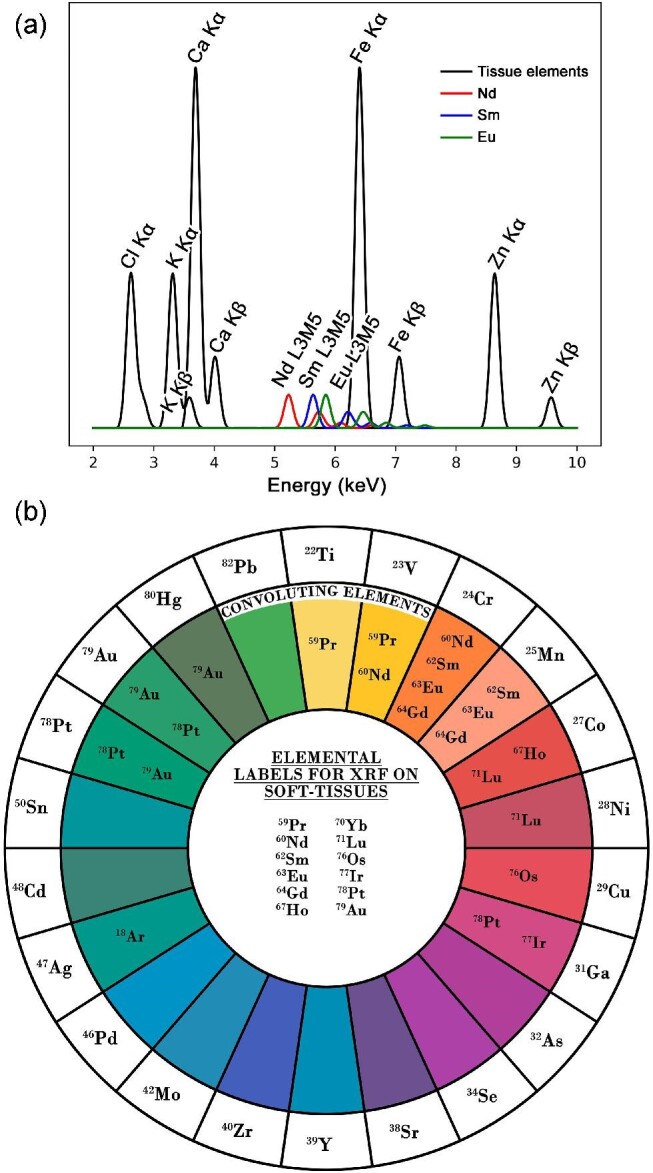
A simulated XRF spectrum (a) shows peak positions of endogenous tissue elements Cl, K, Ca, Fe, and Zn with three candidate element tags (Nd, Sm, and Eu). (b) Illustrates which elements tags provide a fluorescence signal that is discrete from the endogenous tissue signal (central white region). (b) Also highlights potential sources of convolution (coloured rings) when additional exogenous elements are present (outer white circle). For example, the presence of V would cause convolution of Pr and Nd, and therefore these elements should be avoided as candidate labels.

Here to overcome this issue, a new approach using XRF imaging on tissue samples exposed to antibodies conjugated with lanthanide tags is discussed. The method theoretically enables (1) imaging of biological epitopes simultaneously with exogenous and endogenous elements allowing confident correlative studies, (2) a non-destructive approach enabling subsequent guided analysis of tissue sections (speciation measurements), (3) possibility to image >10 simultaneous epitopes surpassing the limitations of conventional fluorophores, (4) capabilities of ‘super-resolution’ as synchrotron X-ray sources can be manipulated to <50 nm in horizontal and vertical planes,^32^ (5) enables ‘multi-resolution mapping’ whereby exploratory low-resolution images can guide smaller high resolution regions of interest due to the non-destructive nature, and (6) potential for tomographic studies due to high tissue penetration of X-rays. The fluorescence emission of lanthanide L-lines, however, can cause significant peak convolution with endogenous elements and/or other labels, therefore, careful experimental planning is required. Figure 4a shows how XRF simulations were used to identify appropriate elemental labels for soft-tissue applications and highlighted areas of expected convolution with other exogenous elements. The simulations only considered the predicted fluorescence emission from the sample and did not include pile-up peaks, escape peaks, or scatter peaks. Pile-up peaks may be a source of convolution if elements with lower masses than the label are highly concentrated. Scatter and escape peaks are dependent on the incident X-ray energy and the type of detector used, respectively. These parameters can be closely controlled to prevent convolution. Due to the expected convolution and lack of isotopic sensitivity a significantly lower number of lanthanide tags can be used in XRF (>10) imaging approaches oppose to IMC (>40) (illustrated in Fig. 4b).^24^ Whilst this limitation may deter certain application where a high number of labels are essential (e.g. complex tumour environments) the added benefits aforementioned such as speciation analysis generates significant benefit for many researchers. The potential of this approach is shown in Figs. 2 and 3, where a single lanthanide label (Sm CD45) is used in two repeats on murine tissue sections showing adequate binding efficiencies and detection in both cases. It is worth noting for this study other labels would have been appropriate, including Nd and Eu, however, Sm was chosen due to the availability of a pre-conjugated form to CD45. This labelling approach enabled simultaneous identification of exogenous and endogenous elemental distributions using XRF. Further guided characterization methods were possible (Fig. 3e) revealing the speciation of exogenous materials due to the non-destructive imaging modality, which is essential for understanding toxicity of the stimulus. It must be recognized that the staining protocol may influence the sample due to the necessary washing steps. Certain elements/compounds are more susceptible to movement, and therefore understanding the mobility of the features of interest is essential and running appropriate controls.^29,33^ In addition, it may be assumed that washout of specific elements, due to sample preparation, happens equally on sub-types of tissues meaning experimental groups can be compared pseudo-quantitatively as well as comparing the elemental heterogeneity across a single image. The distribution of exogenous and endogenous elements showed no significant differences between antibody processed tissues and control tissues in this study. The inherent detection sensitivity of IMC systems currently surpasses that of synchrotron XRF techniques, hence the requirement of a relatively high concentration of antibody for staining. It is envisaged the introduction of fourth generation synchrotrons will significantly reduce the sensitivity gap between these techniques due to provision of increased flux. However, availability of resource is also noted as a limitation. The antibodies used in this study were also optimized for IMC systems and future development of these tags specific for XRF may improve detection capabilities. Using secondary antibodies or increasing the concentration of atoms bound with the antibody will improve the fluorescence signal.

## Conclusions

Investigating correlative relationships between exogenous and endogenous elemental distributions and cellular compositions in tissues is important to help understand their role in health and disease. Multiple discrete imaging approaches are routinely required to study these relationships often necessitating the use of serial sections. Here we have shown, using optical microscopy and confocal synchrotron XRF, the significant heterogeneity in distribution of exogenous particles and cells between immediately adjacent serial sections could result in significant error in 2D correlations and misinterpretation of the data. These data highlight the need for synchronous imaging strategies for correlative studies. We propose a new approach using synchrotron XRF on tissue sections stained with lanthanide conjugated antibodies. This promising non-destructive approach enables simultaneous detection of biological epitopes with the element composition in a highly multiplexed manner at length scales surpassing the diffraction limit of light. We report how element candidates for labels can be selected considering the challenges of fluorescence convolution with endogenous and exogenous elements. Tissue sections lanthanide label (Sm—CD45) showed adequate binding efficiencies and detection in both cases showing the applicability of this technique, although it is recognized that significant developments both of reagents and in detection optimization are required to advance this approach. However, we propose its advantages over conventional correlative imaging strategies to be to quantitatively assess spatial and chemical relationships between sample elements and biological epitopes with greater confidence.

## Materials and methods

### Ex-vivo tissue preparation

Peri-implant tissues were acquired during scheduled revision surgery at the Queen Elizabeth Hospital, Birmingham on patients with short-terms failures (<8 weeks) of bone anchored hearing aid devices (BAH). The inflamed tissue was surgically removed from consenting participants from tissues surrounding the percutaneous commercially pure Ti implant. Tissues were embedded into paraffin and stored at the University Hospital Birmingham. Ethical approval was granted through the University of Birmingham Human Biomaterials Resource Centre (REC 09/H1010/75). Tissue sections were prepared using a conventional microtome with a tungsten carbide blade. Thirty serial sections were prepared at 3 µm and mounted onto charged glass slides (SuperFrost PLUS, Thermo Scientific, USA). The sections were subsequently dewaxed in a xylene and a series of ethanol gradients and exposure to a counter stain of haematoxylin. A thicker section (∼100 µm) was prepared and mounted onto an ultrapure-fused silica slide (Spectrosil 2000, Heraeus Quarzglas GmbH & Co, Germany) for confocal X-ray fluorescence spectroscopy measurements.

### Murine tissue preparation

Male C57BL/6 J mice (Jackson Laboratories, USA), 12–16 weeks of age, were exposed to thiolglycollate to cause predictable inflammation within the peritoneum and anatase nanoparticles (<50 nm) as the environmental Ti exposure.^34^ Saline containing 4% thiolglycollate (Sigma, 70 157, Canada) and 10 ppm suspended TiO_2_ (Sigma, 637 253) nanoparticles were infused intraperitoneally, after which the mice were left for 8 h before sacrifice. Animal studies were approved by the University of Alberta Health Sciences Animal Welfare Committee. Murine spleens and livers were immediately fixed in neutral buffered formalin (10%, Sigma, HT501128, Canada), embedded in paraffin, sectioned at 3 µm and mounted onto ultra-pure fused silica slides.

### Optical microscopy imaging

All slides were imaged using NanoZoomer 2.0 HT Whole Slide Image (Hamamtsu, Japan) using a ×40 objective lens with a resolution of 0.23 µm/pixel in a bright field set-up.

### Confocal X-ray fluorescence spectroscopy

Confocal XRF measurements were performed at the microfocus spectroscopy beamline I18 at Diamond Light Source (Harwell Science and Innovation Campus, UK). An incident energy of 5.7 keV was chosen using a liquid nitrogen cooled Si(111) monochromator and a 5×5 µm X-ray beam was achieved using a Kirkpatrick-Baez focusing mirror system.^35^ The sample was positioned 45° to the incident X-rays and 45° to the fluorescence detector. A half lens polycapillary (XOS, USA) was positioned in front of a Vortex Si drift detector (Hitachi High-Technologies Science, USA) generating a depth profile of 30 µm. An acquisition time of 100 ms per pixel was used to collect fluorescence data in a raster imaging mode. The sample was subsequently translated by 15 µm towards the path of the X-ray beam and imaged. This process was repeated several times to achieve three-dimensional confocal data. XRF data was energy calibrated, batch fitted and outputted as elemental images using software PyMCA (v5.5.5, ESRF).^36^ A mathematical image shift correction was performed on the stacked images, which had occurred due to 45° angle between the sample and X-ray beam. Ti images were threshold at 10x the background and are displayed in a logarithmic scale to help visualize low and high intensity features in a single image. No self-absorption correction is made due to the heterogenous nature of the sample limiting an appropriate attenuation path to be assumed.

### Lanthanide simulations

Simultaneous detection and analysis of endogenous and exogenous elements with the elemental labels using XRF is highly complicated. It is essential that the label used is not present within the sample and the energy required to cause fluorescence is within the same region as the endogenous tissue elements. For this reason, heavy metals, e.g. lanthanides serve as ideal candidates in which their L-shell electrons are excited. However, excitation of a single lanthanide L shell can result in the emission of >20 fluorescence photons with varying wavelengths. Most contain emission probabilities of <1%; therefore, typically only four to six are detected within the data.^37^ Fitting fluorescence peaks is critical for accurate qualitative and quantitative analyses but is complicated by the convolution of data. Therefore, X-ray simulations were performed to identify suitable elements for use as antibody labels. Figure 4a shows an example of an XRF simulation of soft tissue with three labels. The K-shell emission of tissue elements, including Cl, K, Ca, Fe, and Zn, is shown with high intensities as it is expected that they will be present in relatively high concentrations. The largest emission probability from the lanthanide L shell is the L3M5 lines, which are labelled accordingly. These peaks are the most important to not have convolution with tissue or exogenous elements or with other labels. Using XRF simulations, 12 appropriate elements, which have been previously used as labels, have been identified and are displayed in Fig. 4b. Sources of fluorescence convolution are also listed when additional elements are expected to be present within the sample. For example, if Cr is expected to be present, Nd, Sm, Eu, and Gd are not recommended to be used.

### Murine tissue preparation with lanthanide antibodies

Tissue sections were deparaffinized using xylene and rehydrated in decreasing gradients of ethanol before rinsing with distilled water. For heat-induced epitope retrieval, tissues were immersed in sodium citrate buffer (100°C) for 20 min and allowed to cool. The slides were subsequently rinsed with modified phosphate-buffered saline (PBS) (Tween 20) for 2 min twice. For staining, the slides were rinsed in Dulbecco's phosphate-buffered saline (DPBS) for 5 min at room temperature (three times). Bovine serum albumin (10% from powder) was mixed in DPBS and 500 µl was added to each tissue section for 45 min, to prevent non-specific antibody binding. All consumables were sourced from Sigma, Canada. A 1:40 samarium (Sm) anti-mouse CD45 (30-F11, MaxPar®, Fluidigm, USA) in double distilled water was generated and incubated on the sample for 8 h at room temperature. The sample was subsequently rinsed for 5 min with double distilled water and left to air dry for 20 min.

### XRF on lanthanide-stained tissues

Measurements were conducted at I18 (Diamond Light Source) and the X-ray microscopy beamline ID21 (European Synchrotron Radiation Facility). At I18, an incident monochromatic X-ray beam of 7.7 keV was selected and focused to a beam size of 1.5 µm. An irradiation time of 0.1 s per point was used with the fluorescent detector positioned 45° to the sample. Relatively large areas were initially imaged using a 50 µm spot size to identify regions of interest before mapping with a 1.5 µm beam. The samples were measured in air. At ID21, an incident energy of 6.9 keV was used, selected using a fixed-exit double Si(111) crystal monochromator.^38^ The X-ray beam was focused to 500 nm beam size and the tissue imaged under vacuum. An irradiation time of 0.1 s per point was used, and fluorescence emission was collected by an XFLASH 5100 Si drift detector (Bruker, USA). Both data sets were batch fitted, corrected for matrix effects, and outputted as relative counts using PyMCA (v5.5.5).^25^ Qualitative maps displayed were generated using Python 3.0 and matplotlib modules.

X-ray absorption near-edge structure (XANES) measurements were undertaken within an isolated feature of Ti identified by XRF images. XANES measurements were recorded in a fluorescence geometry at ID21 using the previously mentioned set-up. An irradiance time of 0.1 s per energy was used with a total of 300 points over an energy range of 4.9–5.2 keV using variant energy point densities. Data were normalized with the software package ATHENA (v0.9.26)^39^ and compared with Ti standards (metallic Ti, anatase, rutile, and an amorphous Ti oxide complex).

## Data Availability

Data and analysis pipelines can be made available upon reasonable request.
